# CD49f Is an Efficient Marker of Monolayer- and Spheroid Colony-Forming Cells of the Benign and Malignant Human Prostate

**DOI:** 10.1371/journal.pone.0046979

**Published:** 2012-10-12

**Authors:** Hidekazu Yamamoto, John R. Masters, Prokar Dasgupta, Ashish Chandra, Rick Popert, Alex Freeman, Aamir Ahmed

**Affiliations:** 1 Prostate Cancer Research Center, Division of Surgery and Interventional Science, University College London, London, United Kingdom; 2 Department of Urology, Guy's and St. Thomas' NHS Foundation Trust, Great Maze Pond, London, United Kingdom; 3 MRC Centre for Transplantation, King's Health Partners, Guy's Hospital, London, United Kingdom; 4 Department of Histopathology, Guy's and St. Thomas' NHS Foundation Trust, London, United Kingdom; 5 Department of Histopathology, University College London Hospital, London, United Kingdom; The University of Texas M.D Anderson Cancer Center, United States of America

## Abstract

Stem cells may play a role in the development and maintenance of proliferative diseases of the prostate such as prostate cancer and benign prostatic hyperplasia. Cell membrane protein markers, CD49f, CD133 and CD44, have been shown to identify putative prostate stem cells, but a lack of consensus exists with regards to the most efficient marker(s) for stem-like cell identification. This study aimed to determine whether previously reported markers had equal capacity to select monolayer and spheroid colony-forming cells (CFCs), which were used as surrogate readouts of stem-like cells, and to characterize the expression of CD49f, CD44 and CD133 by flow cytometry and immunohistochemistry.

In benign prostate cells, CD49f+, CD44+, and CD133+ cells represented 5.6±3.1%, 28.2±4.1% and 0.10±0.06% of total cells. Both monolayer- and spheroid-CFCs existed at a frequency of approximately 0.5% of total cells. CD49f+, CD44+, and CD133+ subpopulations differed significantly in their ability to select benign CFCs. The highest recovery of CFCs was achieved by CD49f+ selection (98%), whereas CD44+ or CD133+ selection led to poor CFC-recovery (17% and 3%, respectively). For the first time, we show highly efficient recovery of CFCs from advanced prostate cancer by CD49f+, but not by CD44+ or CD133+ selection. Furthermore, CD133 expression (AC133 clone) could not be detected in benign prostate cells by either immunohistochemistry or flow cytometry. We conclude that CD49f, but not previously described stem cell markers CD133 and CD44, to be optimal for selection of monolayer- and spheroid-CFCs in the benign and malignant prostate.

## Introduction

Dysfunctional prostate stem cells are thought to drive the development and progression of proliferative diseases of the prostate such as prostate cancer and benign prostatic hyperplasia [1]–[3]. The paradigm states that specific eradication of abnormal stem cells could lead to better treatment of these conditions [2], and methods to deliver targeted therapies against specific subpopulations of cells already exist [4].

Targeting abnormal stem cells however requires knowledge of specific marker proteins expressed in the subpopulation. Previous investigations have identified a number of putative markers of prostate stem cells [5]–[7], including CD49f [5], CD44 [6], and CD133 [7], alpha2 integrin [6], and Trop2 [5], although currently there is no consensus on the optimal marker(s) for stem cell identification. Similar markers (CD44, CD133 [8] and CD49f [9]) could also identify stem-like cells in prostate cancer. The current literature also lacks evaluation of markers in advanced prostate cancer, a condition associated with a poor prognosis [10]. It is not known whether marker(s) of prostate stem-like cells in benign tissue differ to those in aggressive cancer tissue.

The aims of this study were to characterize the expression of CD49f, CD44 and CD133 in freshly-isolated cells, and compare the efficiency of each candidate marker to identify monolayer and spheroid colony-forming cells (CFCs). CFCs have been used as *in vitro* surrogates of stem-like cells in benign [5], [11], [12] and malignant prostate cells [8],[9]. Both monolayer-CFCs [11], [13], [14] and spheroid- CFCs [5], [15] demonstrate many of the properties of stem cells such as self-renewal, proliferation, three-dimensional gland-formation, and multipotency [11], [13], [16], [17]. In contrast to *in vivo* tissue regeneration assays, colony-forming assays allow enumeration of CFCs within a cell population by colony counts [5], [11], [13], [18]. Here, fresh prostate tissue was enzymatically dissociated into a single cell suspension and labelled with antibody for immunomagnetic cell separation. Subsequently, monolayer- and spheroid-colony-formation assays were used for measurement of CFC yields in fractionated cells. For the first time, we evaluate putative stem cell markers in tissues obtained from patients with at least locally-advanced prostate cancer. Our results indicate significant differences in the capacity of each marker to identify CFCs; and we demonstrate that selection for CD49f+ cells has the highest efficiency of CFC isolation in both the benign and malignant prostate.

## Results

### Flow cytometric characterization of CD49f+, CD44+ and CD133+ subpopulations in cells isolated from benign human prostate tissue

**Figure 1:**
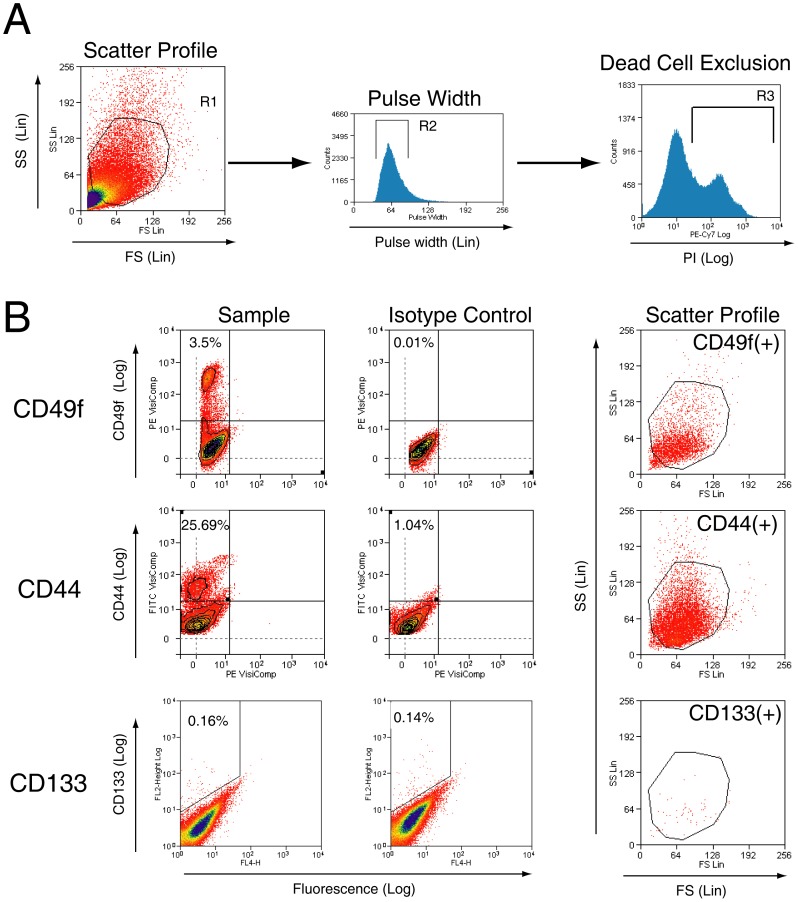
Flow cytometric live-cell analysis of freshly-isolated prostate cells for the identification of CD49f+, CD133+ and CD44+ subpopulations. [**A**] Sequential gating is used to exclude debris by positive selection of scatter gate R1, select single cells by positive selection of pulse width gate R2, and exclude dead cells by negative selection of propidium iodide-positive gate R3. [**B**] Representative dot plots of prostate cells labelled with each antibody. Scatter profiles of prostate cells expressing CD49f, CD44 or CD133 are also shown on the right. FS = Forward scatter, SS = Side scatter.

Flow cytometry was used to determine the proportions of benign prostate cells expressing CD49f, CD44 (clone G44-26) and CD133 (clone AC133). Scatter gating and propidium iodide was used to exclude debris and dead cells arising from tissue digestion (Figure 1A). The specificities of each antibody were validated by positive and negative controls (Figure S1). CD49f+, CD44+, and CD133+ cells represented 5.6±3.1% (n = 5), 28.2±4.1% (n = 3) and 0.10±0.06% (n = 5) of total cells, respectively. Although CD133+ cells could be detected, cellular proportions were not significantly different to that of the isotype control samples (n = 3, p = 0.74) (Figure 1B).

### Monolayer and spheroid CFCs of the benign human prostate

**Figure 2:**
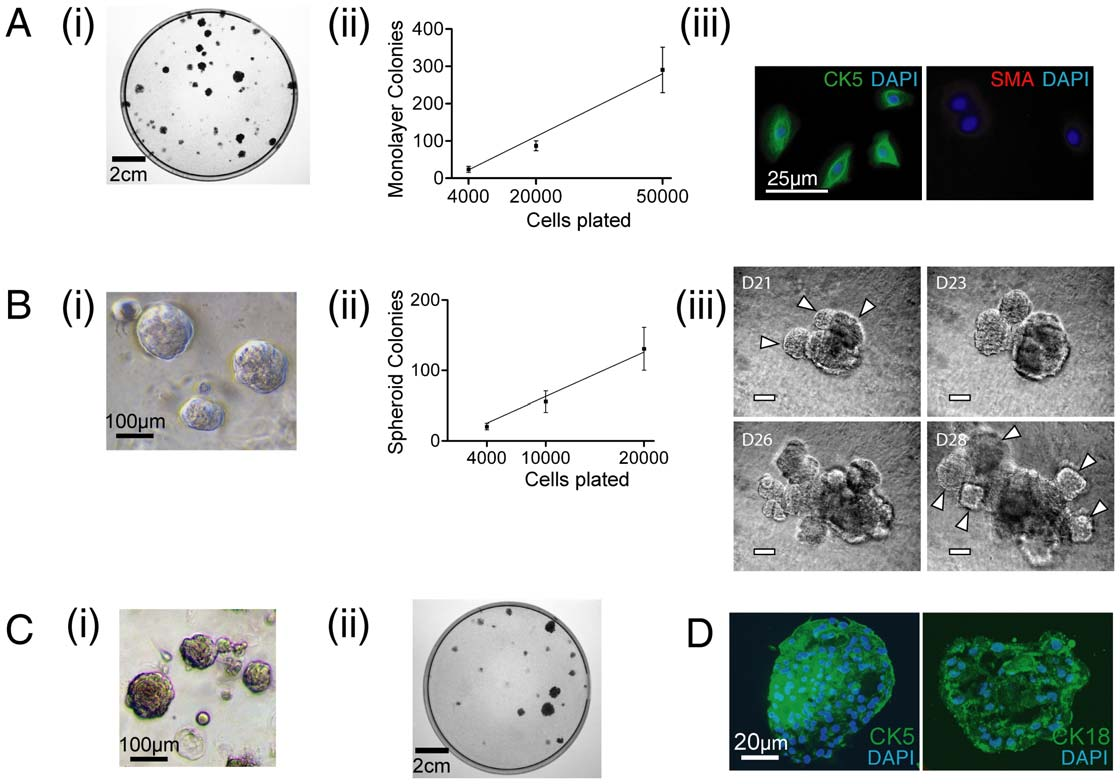
Characterization of monolayer- and spheroid-CFCs. [**A**] (**i**) A representative monolayer colony formation assay on Day 12 is shown. (**ii**) The frequency of monolayer-CFCs within unsorted cells was 0.42±0.07% (n = 5). (**iii**) Cells within monolayer colonies expressed cytokeratin 5 (an epithelial cell marker [20]), but no smooth muscle actin (a stromal cell marker). [**B**] (**i**) A representative image of prostate spheroids on day 12. (**ii**) The frequency of spheroid-CFCs within unsorted cells was 0.45±0.08% (n = 4). (**iii**) Spheroids when kept in culture developed further branching buds (indicated by white arrowheads on day 21 and 28), suggestive of branching ductal structures. [**C**] To show that monolayer- and spheroid-CFCs could represent the same population of cells, cells from monolayer-colonies were used to develop spheroid colonies (**i**), and cells isolated from spheroids were used to form monolayer colonies (**ii**). [**D**] Spheroids expressed markers of both basal (CK5) and luminal (CK18) epithelial cells. CFC = colony-forming cell, CK5 = cytokeratin 5, SMA = smooth muscle actin.

Prostate stem cells, in the mouse, are capable of generating both monolayer- and spheroid-colonies *in vitro* [11]. To demonstrate the colony forming ability of human prostate cells, freshly-isolated human prostate cells were seeded, in parallel, in monolayer- and spheroid-colony-formation assays as previously described [11], [19]. Representative images for each assay are shown (Figure 2A, B). Colony forming efficiencies (CFE, see methods) for monolayer- and spheroid-colonies were similar between assays (0.56±0.06% (Figure 2A), and 0.63±0.8% (Figure 2B), respectively). Colony-derived cells demonstrated an epithelial morphology, and expressed cytokeratin 5 (CK5) [20] but not smooth muscle actin (Figure 2C). When spheroids were enzymatically dissociated and re-seeded, spheroid-forming capacity was retained for up to 4 generations (n = 3), beyond which no further spheroid development was observed. Time-lapse observations during prolonged culture indicated that spheroid colonies could generate further colony buds, suggesting formation of branching ductal structures (Figure 2B). To determine whether monolayer- and spheroid-colonies are derived from the same population, cells were exposed to alternating monolayer and spheroid-colony forming conditions (Figure 2C). This showed that cells within monolayer-colonies could generate spheroid-colonies, and cells within spheroid-colonies could generate monolayer-colonies (Figure 2C, n = 3). Immunohistochemical analysis for multipotency indicated spheroids to express lineage markers of both basal (CK5) and luminal cells (CK18) (Figure 2D).

### Marker identification for monolayer- and spheroid CFCs

To compare the efficiency of cell surface markers in identifying CFCs ‘CFC-recovery’ was measured for each marker (see methods). First, a magnetic-assisted cell separation (MACS) step was used to isolate marker positive (CD49f+, CD44+, and CD133+), and negative (CD49f−, CD44−, and CD133−) cell populations. Technically, MACS yielded similar percentages of positively-labelled cells to flow cytometric analysis, and resulted in efficient recovery of control cells (PC3 for CD49f and CD44, Caco-2 for CD133) (Figure S2). Average post-sort flow cytometric purities of each fraction ranged between 81.4% and 95.4% (Figures S3 and S4).

**Figure 3:**
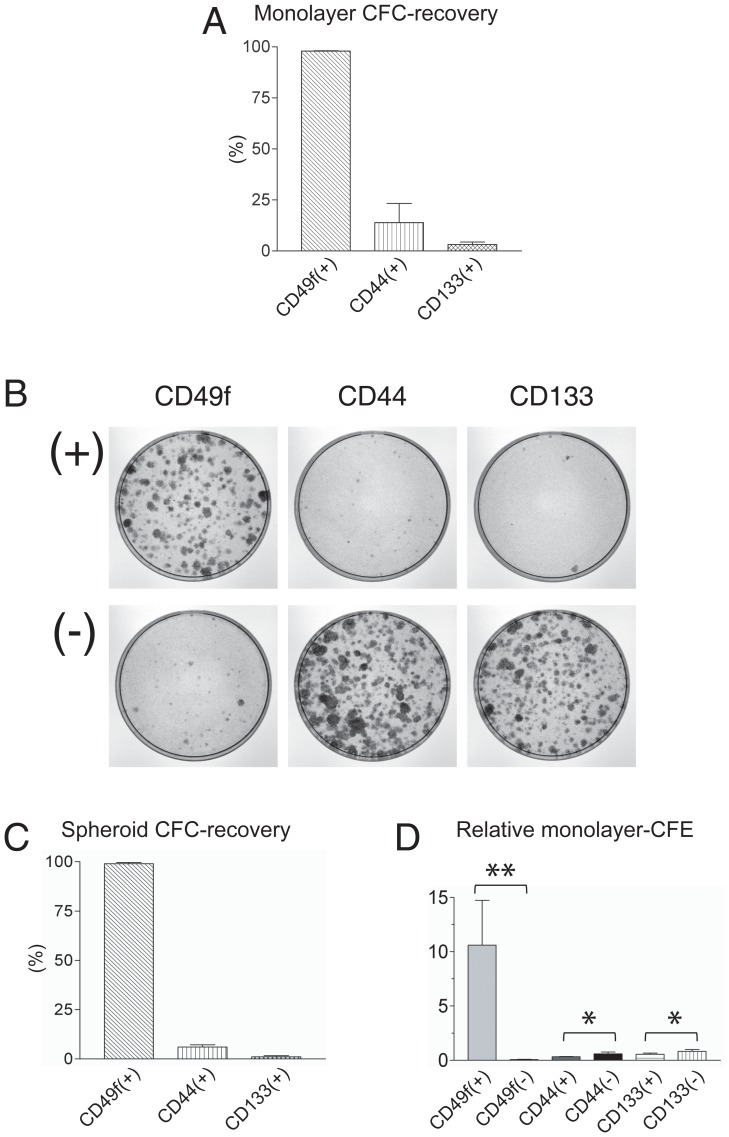
Comparison of markers for selection of monolayer- and spheroid-CFCs. [**A**] Following MACS, total monolayer-colonies arising from each (+)ve and (−)ve fraction were counted to determine the monolayer CFC-recovery, i.e. proportion of input CFCs which are fractionated to the (+)ve fraction (n = 3). The CD49f+ fraction contained 97.9±0.3% of monolayer-CFCs, in contrast to CD44+ and CD133+ cell fractions which contained 13.9±17.9% and 3.1±1.9%, respectively. [**B**] A typical monolayer CFC-assay is shown. (+)ve and (−)ve fractions were derived from immunomagnetic sorting of 50,000 cells, each plated onto 10 cm culture dishes. [**C**] Following MACS, total spheroid-colonies arising from each (+)ve and (−)ve cell fractions were counted to determine the spheroid CFC-recovery (n = 3). The CD49f+ fraction contained 98.9±1.1% of spheroid CFCs, in contrast to CD44+ and CD133+ cell fractions which contained 5.7±2.1% and 0.7±0.6%, respectively (p<0.001). [**D**] CD49f+ were 10.6 fold more enriched in CFCs compared to unsorted cells, and significantly more enriched in CFCs compared to CD49f− cells (p<0.05) (n = 6). No significant CFC enrichment was detected upon comparison of CD44+ and CD44− cells (*n* = 5), or between CD133+ and CD133− cells (n = 3), respectively. ** p<0.05, * p>0.05.

The greatest monolayer CFC-recovery was observed in the CD49f+ fraction (97.9±0.3%, Figure 3A). Monolayer CFC-recovery rates for CD44+ and CD133+ fractions were significantly lower (13.9±17.9% and 3.1±1.9%, respectively, Figure 3A). Therefore, the monolayer CFC-yield following CD49f+ selection was 7-fold and 33-fold than CD44+ or CD133+ selection, respectively (Figure 3B). Results were similar for spheroid CFC-recovery, demonstrating the highest spheroid CFC-recovery in the CD49f+ fraction (98.9±1.1%), whereas CD44+ or CD133+ cells recovered 5.7±2.1% and 0.7±0.6%, of total CFCs, respectively (Figure 3C). Therefore, the spheroid CFC-yield following CD49f+ selection was 17-fold and 140–fold higher than CD44+ or CD133+ cells, respectively, from the same number of unselected cells. Measurement of relative CFE also showed only CD49f+ selection to significantly enrich for monolayer CFCs relative to unsorted cells, with a 10.5-fold relative enrichment (Figure 3D).

### The AC133 epitope is undetectable by immunohistochemistry

**Figure 4:**
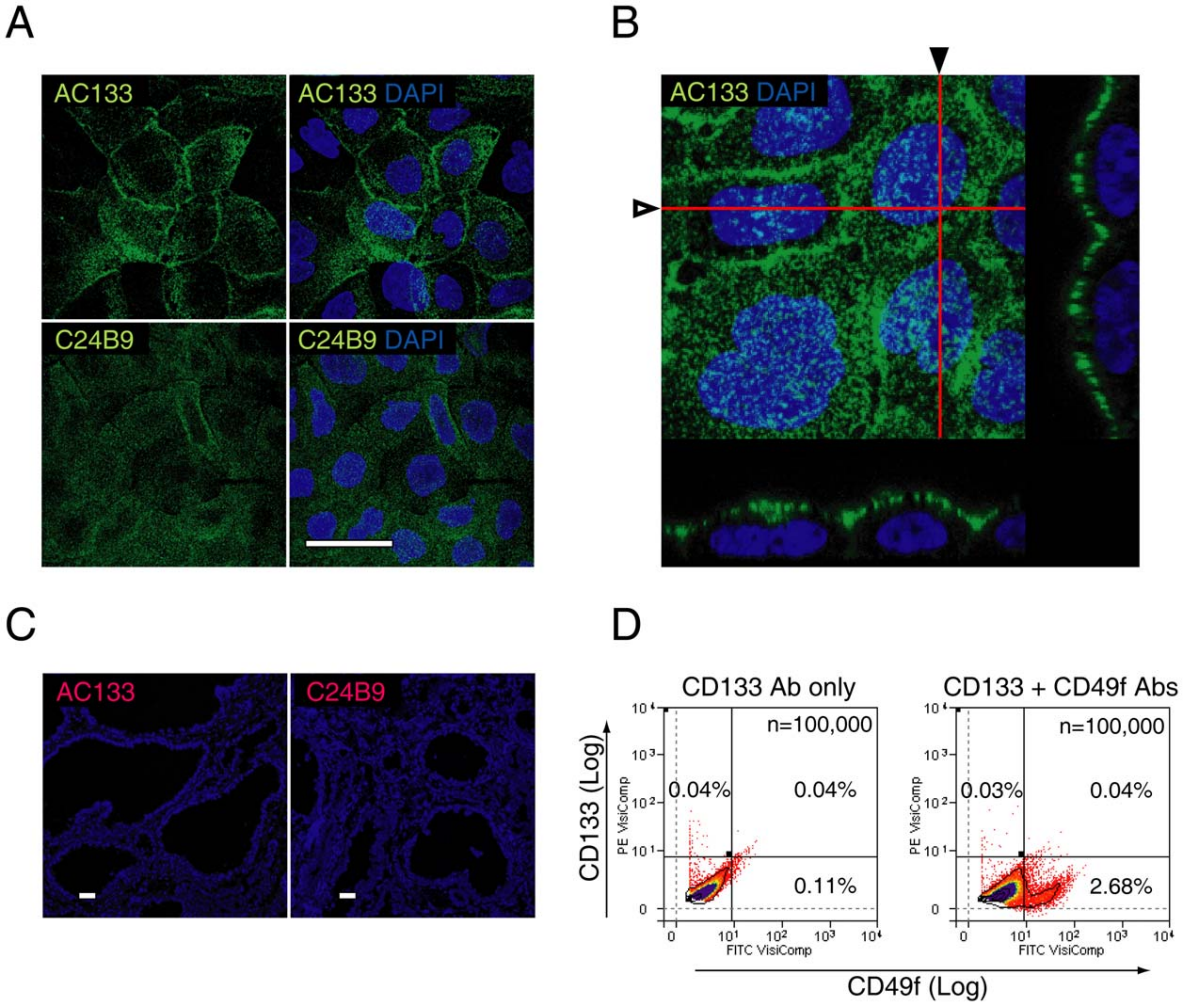
CD133 expression in frozen prostate tissue sections. Validation of CD133 (clones AC133 and C24B9) antibody specificity and expression in the human prostate. [**A**] Punctate expression was shown on the cell surface of Caco-2 cells for clones AC133 and C24B9, as described previously [21]. [**B**] Orthogonal sectioning following three-dimensional reconstruction of 150 slices (red lines marked by hollow and solid arrowheads indicate the x-z planes shown below, or to the right of the confocal image, respectively) indicate CD133 expression only along the apical border of the plasma cell membrane [21]. [**C**] Immunohistochemical expression of AC133 and C24B9 in prostate tissue. Each frozen tissue section measured 10×10 mm in cross-sectional area. Following examination of 20 slides each from 5 patients, we found no cell with definitive membrane expression. [**D**] Flow cytometric co-expression analysis of CD133 and CD49f. A representative flow cytometric analysis of 3 patients shows that CD133+ cells and CD49f+ cells are mutually exclusive populations, with no significant increase in the percentage of cells within the CD49f+/CD133+ cell gate compared to control. Scale bar = 20 µm.

The results described above indicated that AC133 clone-selected CD133+ cells lacked statistically significant colony-forming capacity (Figure 3). To further assess the protein expression of CD133, immunohistochemistry was conducted using frozen prostate sections and two CD133 antibody clones, AC133, and C24B9. Antibody specificities were validated using known positive controls (Caco-2 and HT29 cell lines) [21], which confirmed punctuate, apically polarised expression of CD133 (Figure 4A, B), as described previously [21]. Surprisingly, no immunohistochemical evidence of CD133 expression was found for either clone in 100 slides of hemi-prostate sections (Figure 4C).

To further characterize the CD133+ population, we determined its expression relative to CD49f, a marker that we found to be most selective for CFCs. Dual-label analysis conducted by flow cytometry showed CD133+ cells and CD49f+ cells to be independent cell populations (n = 3) (Figure 4D).

### Further characterization of CD49+ cells of the benign human prostate

**Figure 5:**
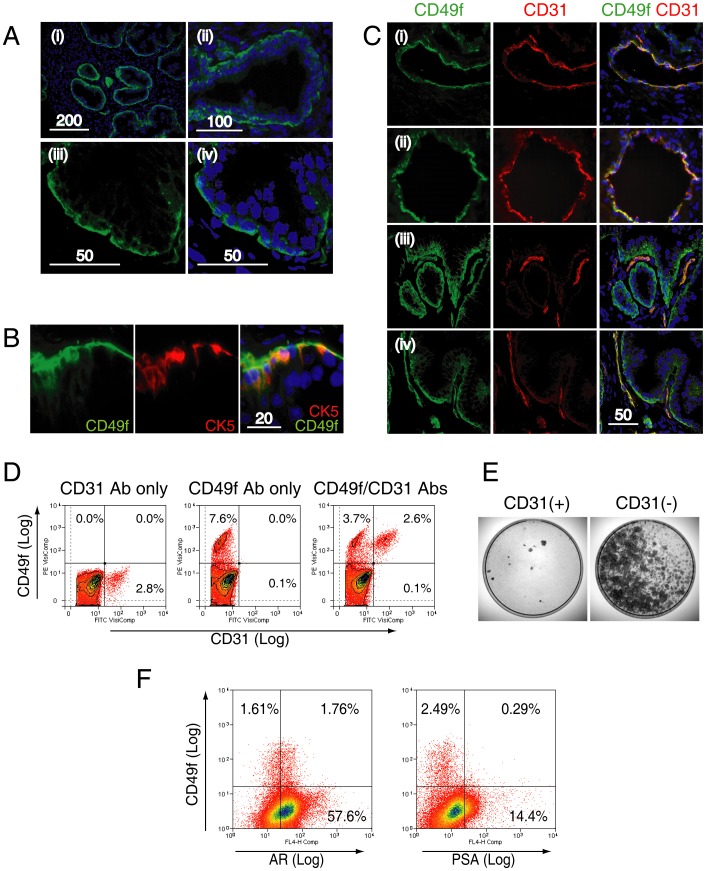
Characterization of CD49+ cells in the benign prostate. **A.** CD49f expression was assessed in frozen sections of prostate tissue at low (i), medium (ii) and high magnification (iii, iv) by confocal microscopy. Expression was polarised towards the outer surface of the basal cell layer as reported previously (iii, iv) [22]. **B.** Co-expression of CD49f with CK5, a basal cell specific marker. **C.** CD49f expression was also found in endothelial cells as demonstrated by co-expression with CD31, a pan-endothelial cell marker. Endothelial cells formed either a luminal (rows (i) & (ii)), or a linear structure (rows (iii) & (iv)) within the stroma. Co-localization of CD31 with CD49f (indicated by yellow color) was only observed in the stromal compartment but not in the basal layer. **D.** Human prostate cells labeled with CD31 and CD49f (representative of 3 samples). CD31+ cells alone represented in 3.0±1.5% of human prostate cells. CD31+ cells formed a distinct subpopulation within CD49f+ cells, and all CD31+ cells were CD49f+. **E.** Colony-forming cell assays conducted using CD31+ and CD31− populations conducted by sorting 50,000 cells by MACS (n = 3) showed, in all assays, almost no colonies in the CD31+ fraction. Scale bar = µm. **F.** A representative flow cytometric co-expression analysis of CD49f with androgen receptor (AR) or PSA is shown.

Expression of CD49f (clone GoH3) was observed in the cell membranes of basal cells lining the prostatic acini (Figure 5A). CD49f co-localized with CK5 (Figure 5B), and expression polarised towards the outer surface of basal cells facing the stroma, as previously reported [22] (Figure 5A).

CD49f expression was also found in endothelial cells, as demonstrated by co-localization with CD31 (Figure 5C (i)–(iv)), a pan-endothelial marker [23], [24]. CD49f+/CD31+ cells were arranged either in a luminal (Figure 5C (i),(ii)) or linear configuration (Figure 5C (iii),(iv)), consistent with the normal histological arrangement of endothelial cells (as assessed by a histopathologist). Moreover, CD31+ cells were exclusive within CD49f+ cells, accounting for 3±1.5% of total human prostate cells (n = 3) (Figure 5D). CD31 expression, however, did not identify CFCs as shown by the lack of clonogenic capacity of CD31+ cells in contrast to CD31− cells (n = 3) (Figure 5E). 45.3±5.2% (n = 3) of CD49f+ cells did not express the androgen receptor (AR) [25], and very few were positive for prostate specific antigen (PSA), a marker of luminal cell marker [26]. These results, in combination, indicated that CFCs are likely to be CD49f+/CD31−, basal-like cells, with and without AR expression.

### Selection of CFCs within locally advanced prostate biopsy tissue

**Figure 6:**
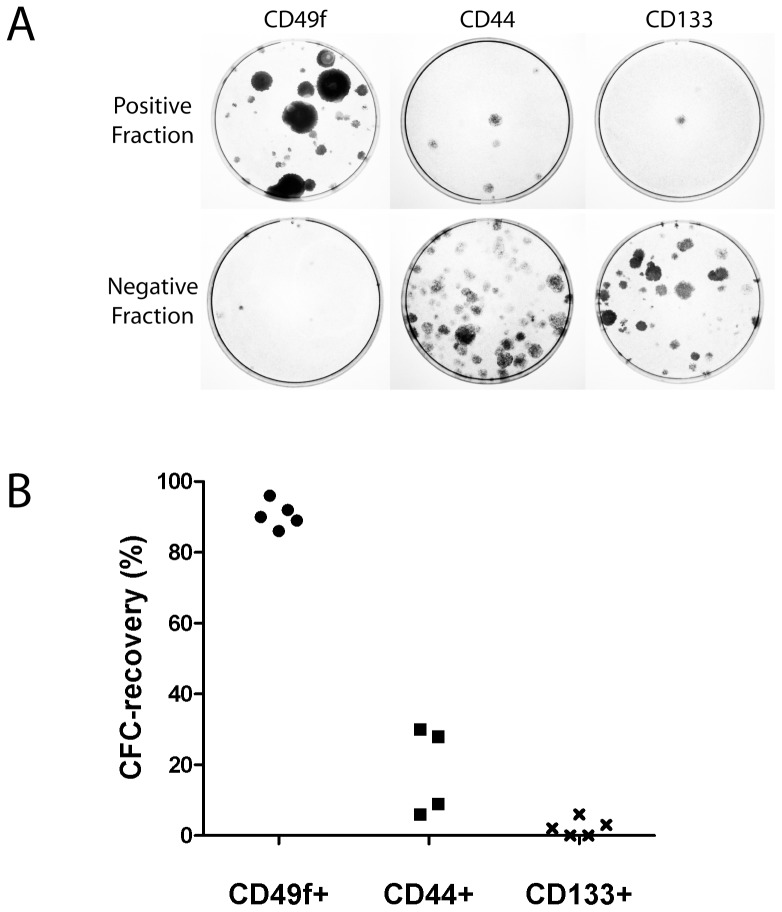
CFC-recovery following CD49f+, CD44+, and CD133+ selection, in advanced prostate cancer. **[A]** A representative monolayer colony-formation assay arising from positive and negative fractions of putative markers is shown. Amongst the positively selected fractions, the greatest numbers of colonies is found in the CD49+ fraction. **[B]** CD49f+ selection recovers the largest number of monolayer-CFCs.

Eight core needle biopsy samples (clinico-pathological characteristics of the five patients are shown in Table 1) were obtained from patients with locally advanced or metastatic prostate cancer, of which five samples survived the combination of cell isolation, MACS, and monolayer colony formation. Tissue was digested using collagenase to give a single cell suspension (in a manner similar to that described for benign tissue, above) and MACS was used to obtain positive and negative fractions using antibodies for CD49f, CD44, and CD133. A representative colony formation assay is shown (Figure 6A). The numbers of colonies arising from each fraction were counted to determine the CFC-recovery (Figure 6). CFC-recovery was highest for CD49f (90.6±3.7%) compared to CD44 (18.3±12.5) or CD133 (2.2±2.5) (Figure 6B), similar to the data obtained from benign prostate cells.

**Table 1:** Clinico-pathological characteristics and CFC-recovery following immunomagnetic cell separation.

Patient	Age	PSA	Prior Treatment	Clinical stage	Gleason/Bone scan mets	No. of cores involved	% core involvement (range)	CD49f CFC-recovery %	CD44 CFC-recovery %	CD133 CFC-recovery %
**1**	81	50	nil	T3	7/N	10/12	30–100	92 (36/39)	9 (6/69)	2 (1/42)
**2**	72	478	nil	T3	8/Y	6/6	80–100	89 (25/28)	30 (10/36)	0 (0/20)
**3**	73	55	AB	T3	8/N	6/12	50–100	96 (25/26)	6 (2/35)	3 (1/40)
**4**	76	66	nil	T3	9/N	11/12	30–90	86 (61/71)	28 (10/35)	0 (0/46)
**5**	68	92	nil	T3	9/N	10/12	20–90	90 (52/58)	*	6 (2/36)
**Mean ±SD**	74±5	148±185						90.6±3.7	18.3±12.5	2.2±2.5

All patients had Gleason score 7 or above, clinical stage T3, and one patient had metastatic disease at presentation. CFC-recovery (defined as the proportion of total CFCs within the positive fraction) was measured for each marker, with CD49f demonstrating the highest recovery.

AB = androgen ablation, SD = standard deviation.

* sample infected.

## Discussion

A direct *in vitro* comparison of cell surface markers, CD49f, CD44, and CD133, showed CD49f to select CFCs with the highest efficiency in both benign and malignant prostates. CD49f expression also identifies the cell of origin of prostate cancer [27], [28], and the tumor-initiating subpopulation in a prostate cancer model [9]. Our results indicate that CD49f expression identifies CFCs within high-risk prostate cancer.

CFCs have been used as an *in vitr*o surrogate readout of stem-like cells in a number of adult tissues [11], [13], [29]–[31]. For the prostate, this is supported by the equivalence of markers that identify CFCs and tissue regenerating stem cells [5], [32]. Bonafide prostate stem cell markers remain controversial, however, as genetic lineage tracing experiments have shown that stem cell subpopulations could be defined by other markers [33].

Here, we demonstrate that monolayer- and spheroid-CFCs were rare, both present at a frequency of approximately 0.5% of total prostate cells (Figures 2A, B). CFCs underwent clonal proliferation, generated branching ductal structures (Figure 2B) and expressed both basal and luminal lineage markers (Fig. 2D).

CD49f is an integrin, a receptor with a high binding affinity for collagen as used in our monolayer colony forming assays. An interaction between CD49f and collagen was theoretically possible, however, the lack of collagen coating of culture flasks did not affect the overall result that CD49f optimally selected for CFCs (Figure S5).

Our findings are consistent with previous studies that indicate CD49f as a marker of epithelial stem cells [5], [11], [13],[28]. A striking finding in our study, however, was that the vast majority of CFCs (90–98%) resided within the CD49f+ population, not only in benign but also malignant prostate tissue. This implies that targeting of CD49f+ cells in the malignant prostate would eliminate the vast majority of the clonogenic cell population.

Further characterization also revealed CD49f+ cells to be comprised of two cell types, CK5+ basal epithelial cells, and CD31+ endothelial cells (Figures 5C,D). The fact endothelial cells expressed CD49f is pertinent in the context of cancer treatment, as neovascularization is a critical hallmark of cancer [34]. Targeting CD49f+ cells in prostate cancer could therefore simultaneously eliminate clonogenic cells and abnormal endothelial cells associated with neovascularisation.

We found a lack of AR expression in a subpopulation of CD49f+ cells (Figure 5F), consistent with a study that showed AR expression in a subset of basal epithelial cells [25]. The lack of AR expression is considered a feature of androgen-independence of prostate stem cells [35]. Similar findings are reported in prostate cancer, where stem-like cells with efficient tumor-initiation capacity were found within a subpopulation of the AR−/PSA− cells in CWR22 orthotopic xenografts [36]. The lack of PSA expression as a marker is also reported in a study that showed PSA^−/lo^ cells, in contrast to PSA^+^ cells, to possess higher tumorigenicity and sphere-colony forming capacity in androgen-ablated mice [37].

CD133 is a well-characterized marker for hematopoietic [38] and neural stem cells [39], although controversy exists regarding its utility in other tissues [40]. A previous study showed selection for CD133+ cells resulted in enrichment for monolayer-CFCs, although several pre-selection steps were required immediately prior to CD133+ selection, including differential centrifugation, “basal-like” cell selection, and rapid attachment selection [7]. Another marker, CD44, has been suggested as marker for stem-like cells [41], although until now, direct comparisons of CFC numbers within CD133+ or CD44+ cells, against respective CD133− or CD44− cells, using only a single cell sorting step, have not been performed. We show that CD44+ and CD133+ cells were depleted in CFCs compared to their respective negative populations, in both benign (Figure 3) and malignant prostate cells (Figure 6). These results imply that a single therapeutic agent targeting CD44+ or CD133+ cells in prostate cancer would not target the majority of clonogenic cells.

Our study also demonstrates an approach to compare different sorted subpopulations in parallel, with incorporation of validation assays for quality control. To minimize false positive signals at flow cytometry, we used live cell analysis in conjunction with dead cell exclusion [42] (Figure 1A). The specificities of antibodies were confirmed using well-characterized positive and negative controls to control for non-specific binding (Figure S1). Accuracy of MACS was validated by cell yield and purity analysis (Figures S2, S3, S4). Finally, we also avoided pre-selection steps to ensure that cells were sorted purely according to the expression of a single marker.

We found CD133 to be a poor marker of CFCs using the stem cell-selective antibody clone AC133 [38]. AC133 selected the least number of CFCs in both benign and malignant cases (Figure 3, 6), and AC133 expression was not observed by immunohistochemistry in our frozen sections. Because glycosylation of epitopes may influence the detection of CD133 (AC133) expression [43], immunohistochemistry was also conducted using another commercially available clone C24B9 whose binding is not affected by glycosylation; however, this also failed to detect CD133 expression in prostate tissue sections. By flow cytometry, we found CD133+ cells alone to represent a rare population of around 0.1% within human prostate cells (Figure 1B), similar to previous reports [7], although this percentage was statistically no different to isotype cells. Data for isotype controls were not shown in previous studies of flow cytometric expression analysis of fresh prostate cells [7], [43]. Variability in CD133 expression, however, is well-recognized in other tissues [40], [44], and reports of CD133 expression in the prostate also vary widely from that of no expression [45], to abundant expression in luminal cells [46]. Our results of immunohistochemistry and flow cytometry support findings by Sotomayer et al. [45], which showed AC133 epitope expression to be either too rare to be detected or not present in the prostate.

## Conclusions

We have characterized the expression of putative stem cell markers, CD49f, CD44, and CD133, in benign and malignant prostate cells. Functional assays demonstrate CD49f to be the most efficient marker for identifying the colony-forming cell population within benign prostatic hyperplasia and advanced prostate cancer tissues.

## Materials and Methods

### Ethics statement

Ethical approval was given by the Joint UCL/UCLH committees on the ethics of human research. The review board approved the use of human tissue for prostate cancer research, in compliance with the International Committee on Harmonisation of Good Clinical Practice (ICH GCP).

### Patient consent and tissue collection

Informed written consent was given by each patient prior to prostate tissue acquisition. Benign prostate tissue was obtained from 27 patients undergoing holmium laser enucleation of prostate (HoLEP), to obtain tissue pieces around 1–2 mm in diameter. All HoLEP samples were confirmed as histologically benign by the uropathologist. Clinical characteristics of each patient are shown in Table S1.

Biopsy tissues from clinically locally-advanced prostate cancer were obtained from 8 patients with PSA more than 50 ng/ml, undergoing diagnostic transrectal ultrasound-guided prostate biopsies. An 18-gauge biopsy needle was used to take two core biopsies from each patient, in addition to standard diagnostic biopsies (6–12 cores). Adjacent diagnostic biopsies were sent separately for histopathological correlation. The average wet weight of each biopsy was 6.5±1.23 mg.

### Preparation of a single cell suspension of human prostate cells

The tissue was washed, minced and digested at 37°C in PrEGM solution (Lonza), containing 600 U/ml collagenase IV, 0.4% bovine serum albumin and 100 U DNAse I, for up to 4 h (2 h for biopsy tissue) and filtered through 100 µm and then 40 µm meshes to obtain a single cell suspension. Pharmlyse (BD Biosciences) was added to lyse red cells and remaining cells were counted using trypan blue exclusion. The average viable cell yield from HoLEP tissue was 4.4±3.7×10^6^ cells/prep (range: 0.4–14.1×10^6^ cells, n = 27). For biopsy tissue, two cores from each patient were pooled prior to mechanical and enzymatic digestion, yielding approximately 50,000–80000 cells per patient.

### Flow cytometric analysis

The following antibodies were used for flow cytometric analysis: CD49f (clone GoH3, BD Biosciences), CD44 (clone G44-26, BD Biosciences), and CD133 (clone AC133, Miltenyi Biotec), and CD31 (clone WM59, BD Biosciences). Freshly-isolated cells were incubated with fluorochrome-labelled antibody or isotype control antibody in PBS containing 1% BSA, and analysed using the CyAn™ ADP Analyser (Beckman Coulter). Isotype control antibodies were used to set the positive gates. Forward and side scatter gates, and pulse width selection, were used to exclude small debris, and aggregated cells, respectively (Figure 1A). Propidium iodide was used for dead cell exclusion (Figure 1A) [13]. Multicolor compensation was conducted post-analysis using median intensity values of the control population. 50,000 to 100,000 live, single cell events were recorded per analysis. Bi-exponential scales and isodensity contour lines were used to display dot plots [47].

### Monolayer colony-formation assay

Cells were plated onto dishes pre-coated with 10 µg/ml rat-tail collagen 1 (Sigma-Aldrich) in serum-free Prostate Epithelial Cell Growth Medium (PrEGM) containing penicillin (10 U/ml) and streptomycin (10 µg/ml) [19]. NIH/3T3 cells, treated with Mitomycin C (10 µg/ml), were plated at 15000 cells per cm^2^ to act as a feeder layer. A colony was defined as a cluster of more than 32 cells at day 12 [19].

### Spheroid colony-formation assay

Cells were suspended in a 1∶1 mixture of PrEGM∶Matrigel, and then plated around the rims of wells of a 6 or 24 well plate [11]. PrEGM was added after allowing to each gel to solidify for 15 min. A spheroid colony was defined as a sphere-shaped three-dimensional colony of at least 50 µm in diameter at 12 days. For enzymatic dissociation of sphere colonies into single cells, Matrigel scaffolds were liquefied using dispase to release the spheroid colonies. Spheroid colonies were collected and pelleted in centrifuge tubes and dissociated using collagenase and trypsin, as described previously [11].

### Immunohistochemistry

Frozen tissue sections were used for immunohistochemical analysis. A cryostat was used to obtain 4–8 µm sections measuring approximately 2×2 cm from frozen tissue blocks freshly cut at cystoprostatectomy. Tissues were all confirmed retrospectively as histologically benign. Each frozen section was fixed for 60 s in 70% ethanol, washed three times in PBS, and incubated with 5% BSA for 45 min at RT (room temperature) to block non-specific binding. The following unlabelled primary antibodies were purchased; CD49f (clone GoH3, BD Biosciences), CD31 (clone WM59, BD Biosciences), CD133 (clone AC133, Miltenyi Biotec), CD133 (clone C24B9, Cell Signalling Technology), and used with Alexa-Fluor-conjugated secondary antibodies (Invitrogen). Briefly, samples were incubated overnight at 4°C with primary antibodies, and for 60 min at RT in the dark with secondary antibody. Images were taken using the TCS SPE2 confocal microscope (Leica Microsystems).

### Cell separation

Immunomagnetic cell separation (MACS) was conducted following the manufacturer's protocol (Miltenyi Biotec). Briefly, prostate cells were labeled with flurochrome-conjugated antibodies, washed, and incubated with anti-fluorochrome magnetic nanobeads. Samples were passed through a magnetized MACS MS column to collect the negative fraction. The column was then demagnetized and flushed twice to collect the positive fraction. For determination of post-MACS cell yields, 1×10^6^ cells were sorted in triplicate and viable cell yields were measured by trypan blue exclusion.

### CFC-recovery and Colony-forming efficiency (CFE)

CFC-recovery was defined as: (total colonies arising from the positive fraction)/(total colonies arising from both positive and negative fractions) [48]. To measure CFC-recovery, 50,000 human prostate cells were immunomagnetically sorted based on expression of one marker, and each positive and negative fraction was seeded onto individual 10 cm dishes for monolayer colonies, or in Matrigel® to generate spheroid colonies. Colonies were counted on day 12.

CFE was defined as: (colonies per dish)/(cells seeded per dish). Relative CFE, defined as (CFE of sorted cell fraction)/(CFE of unsorted cells), was used to standardize CFE across samples.

### Data analysis and statistics

Unless otherwise stated, data is presented as mean± s.d. Statistical analyses were performed using the Graphpad Prism software.

## Supporting Information

Figure S1 Evaluation of specificity of CD49f (GoH3), CD44 (G44-26) and CD133 (AC133) antibodies using cell lines. Dead cells and cell doublets were excluded as described in the methods section. Specificities of all three antibodies were confirmed as consistent with previous studies; CD49f was highly expressed in >90% of PC3, LNCaP and DU145 cells [49]; CD44 showed expression in >98% of PC3 cells, <1% of LNCaP, and in a subpopulation of DU145 cells, as previously reported [41], [49]–[51]. CD133 was expressed in >98% of Caco-2 and HT29 cells [21], [43], [52], [53].
(TIF)

Figure S2 Post-MACS cell yields of (+)ve and (−)ve fractions are expressed as a percentage of pre-MACS input cells (n = 3). Positive controls (PC3 for CD49f and CD44, Caco-2 for CD133) were used to evaluate the technical success of immunomagnetic cell separation.
(TIF)

Figure S3 Flow cytometric purity assessment of positive and negative cell fractions following immunomagnetic separation of freshly-isolated human prostate cells using CD49f and CD44 antibodies (*n* = 3 patient samples, R1, R3, and R20, see Table S1). Gates were set using isotype controls of unsorted cells. Purities of the CD49f+ and CD49f− fractions were 81.4±6.6% and 91.1±6.3%, respectively. Purities of the CD44+ and CD44− fractions were 91.5±5.6% and 88.2±2.9%, respectively.
(TIF)

Figure S4 Flow cytometric purity assessment of positive and negative cell fractions following immunomagnetic separation of a mixture of Caco-2 and PC3 cells using CD133 (AC133) antibody (representative images of *n* = 3). **A.** Flow cytometric analysis of unsorted Caco-2 and PC3 cells. Almost all Caco-2 cells express CD133 (>97%), in contrast to PC3 cells which were CD133− by phenotype. **B.** The two cell lines were mixed at a ratio of 2 PC3 : 1 Caco-2 and sorted by MACS to obtain positive and negative fractions. Flow cytometric purities of CD133+ and CD133− fractions post-selection were 95.4±3.21% and 81.7±5.40%, respectively (n = 3). *n* = 20,000 live cells for all analyses. FS = forward scatter. MACS = magnetic cell separation
(TIF)

Figure S5 **A.** Human prostate epithelial cells can be cultured without collagen coating (a representative image of an epithelial colony is shown using the same protocol described in the methods, but without collagen pre-coating). **B.** CD49f is an integrin with potential to bind collagen [54]. To determine whether collagen-coating was responsible for the enhanced colony forming capacity of CD49f+ cells, the colony-forming assay was repeated as described in Figure 3, without collagen pre-coating. In the absence of collagen coating, our results again showed the highest colony-forming cell recovery in CD49f+ cells.
(TIF)

Table S1 Table indicating the sources of prostate tissue, patient age, and PSA value for each benign tissue. All samples listed were histologically confirmed to have benign histology. HoLEP = Holmium laser enucleation of prostate.
(DOC)
