# Supplementary figures and images for: CD49f Is an Efficient Marker of Monolayer- and Spheroid Colony-Forming Cells of the Benign and Malignant Human Prostate
Source: PLoS One. 2012 Oct 12;7(10):e46979. doi: 10.1371/journal.pone.0046979 (PMC3470557; doi:10.1371/journal.pone.0046979)

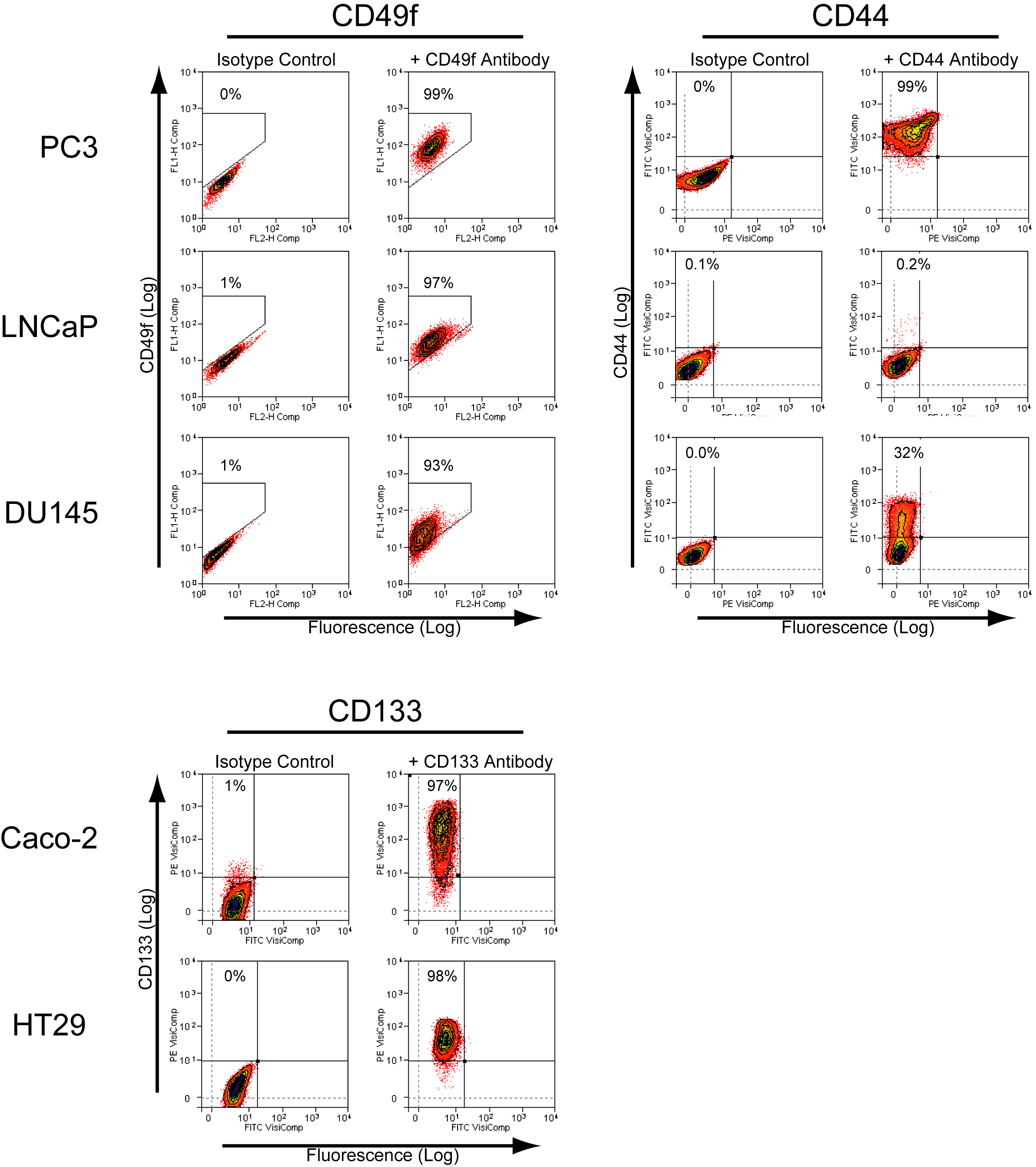

Supplement: Figure S1 — Evaluation of specificity of CD49f (GoH3), CD44 (G44-26) and CD133 (AC133) antibodies using cell lines. Dead cells and cell doublets were excluded as described in the methods section. Specificities of all three antibodies were confirmed as consistent with previous studies; CD49f was highly expressed in >90% of PC3, LNCaP and DU145 cells [49]; CD44 showed expression in >98% of PC3 cells, <1% of LNCaP, and in a subpopulation of DU145 cells, as previously reported [41], [49]–[51]. CD133 was expressed in >98% of Caco-2 and HT29 cells [21], [43], [52], [53]. (TIF) [file pone.0046979.s001.tif]

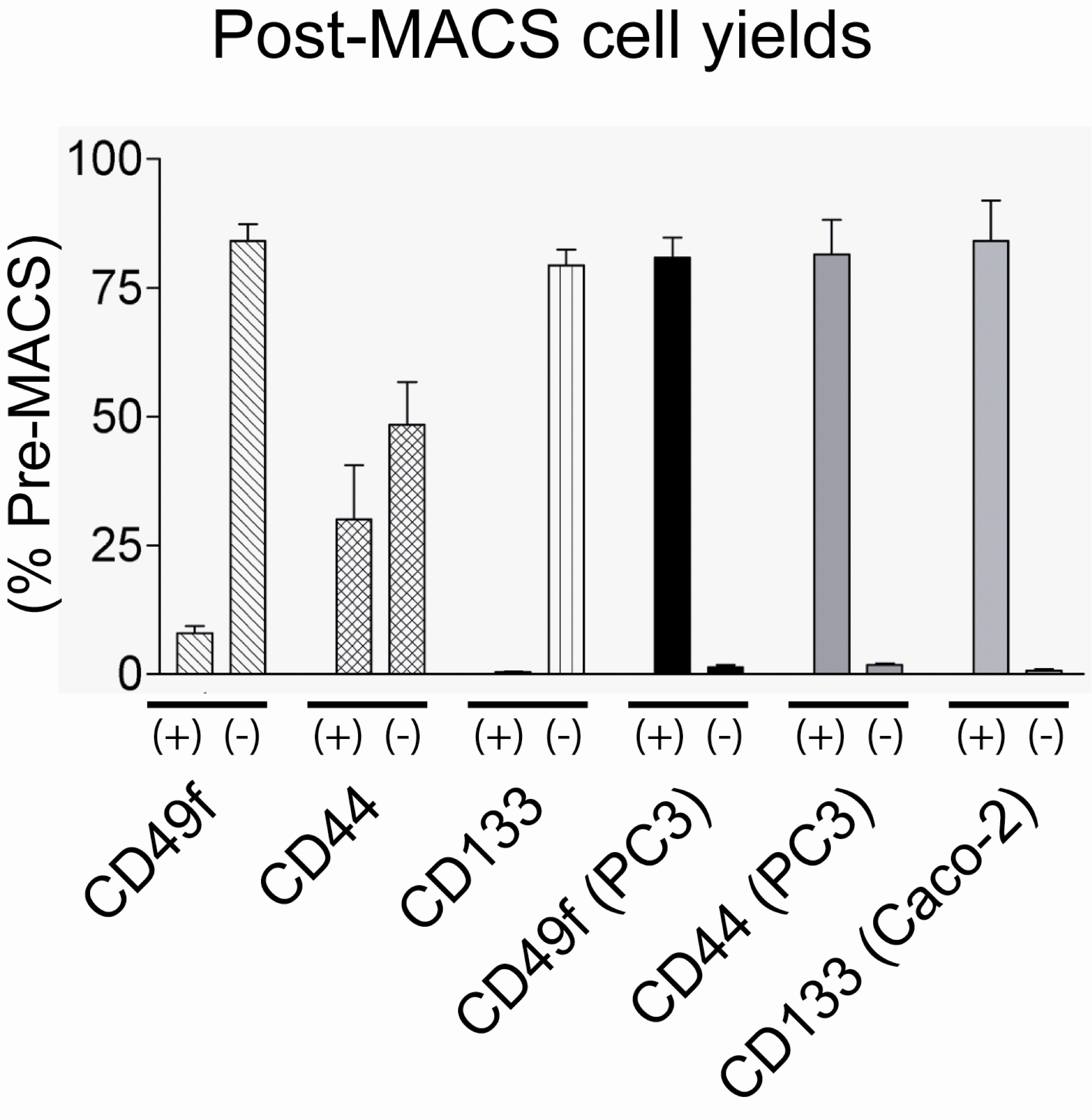

Supplement: Figure S2 — Post-MACS cell yields of (+)ve and (−)ve fractions are expressed as a percentage of pre-MACS input cells (n = 3). Positive controls (PC3 for CD49f and CD44, Caco-2 for CD133) were used to evaluate the technical success of immunomagnetic cell separation. (TIF) [file pone.0046979.s002.tif]

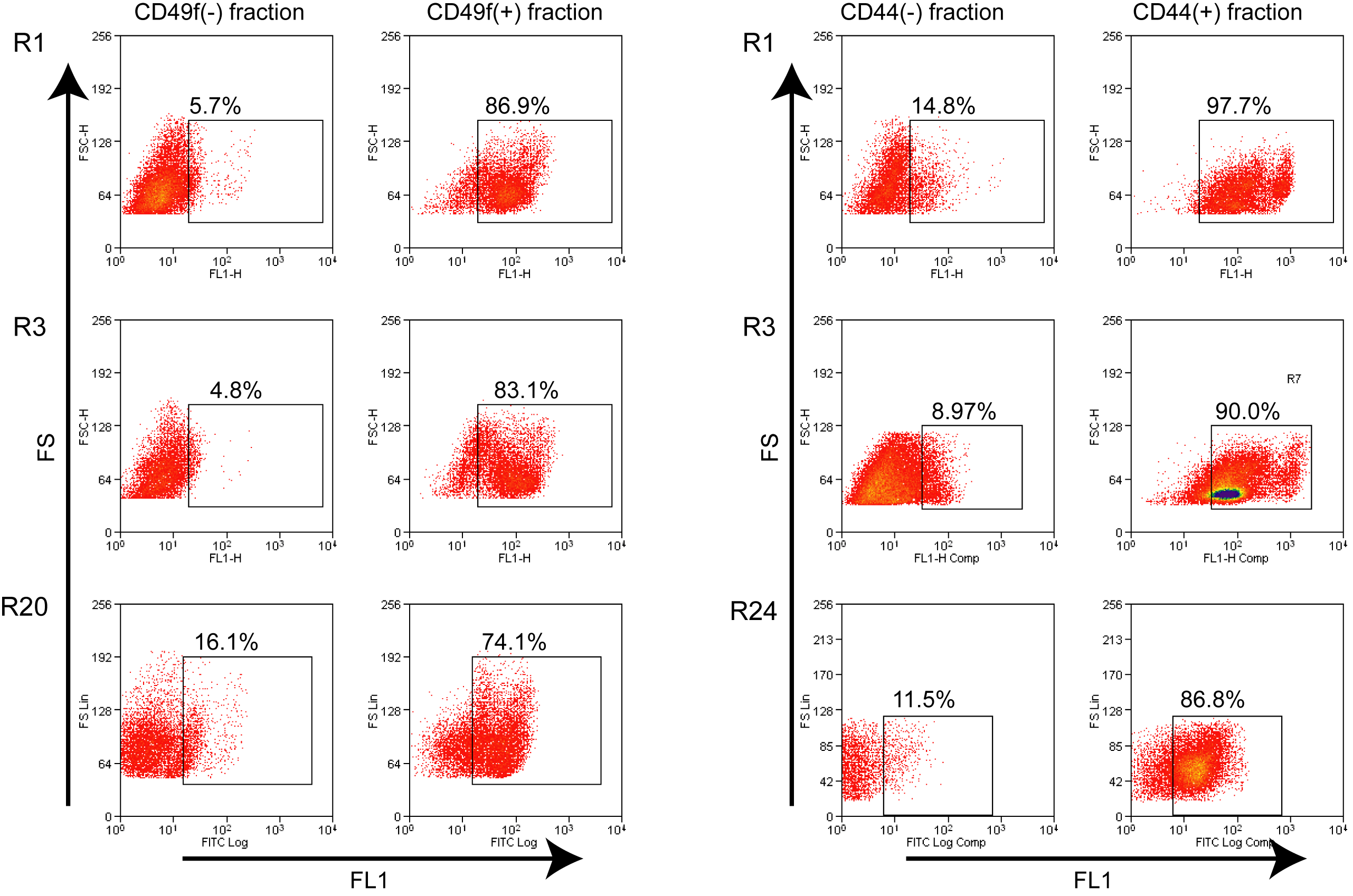

Supplement: Figure S3 — Flow cytometric purity assessment of positive and negative cell fractions following immunomagnetic separation of freshly-isolated human prostate cells using CD49f and CD44 antibodies (n = 3 patient samples, R1, R3, and R20, see Table S1). Gates were set using isotype controls of unsorted cells. Purities of the CD49f+ and CD49f− fractions were 81.4±6.6% and 91.1±6.3%, respectively. Purities of the CD44+ and CD44− fractions were 91.5±5.6% and 88.2±2.9%, respectively. (TIF) [file pone.0046979.s003.tif]

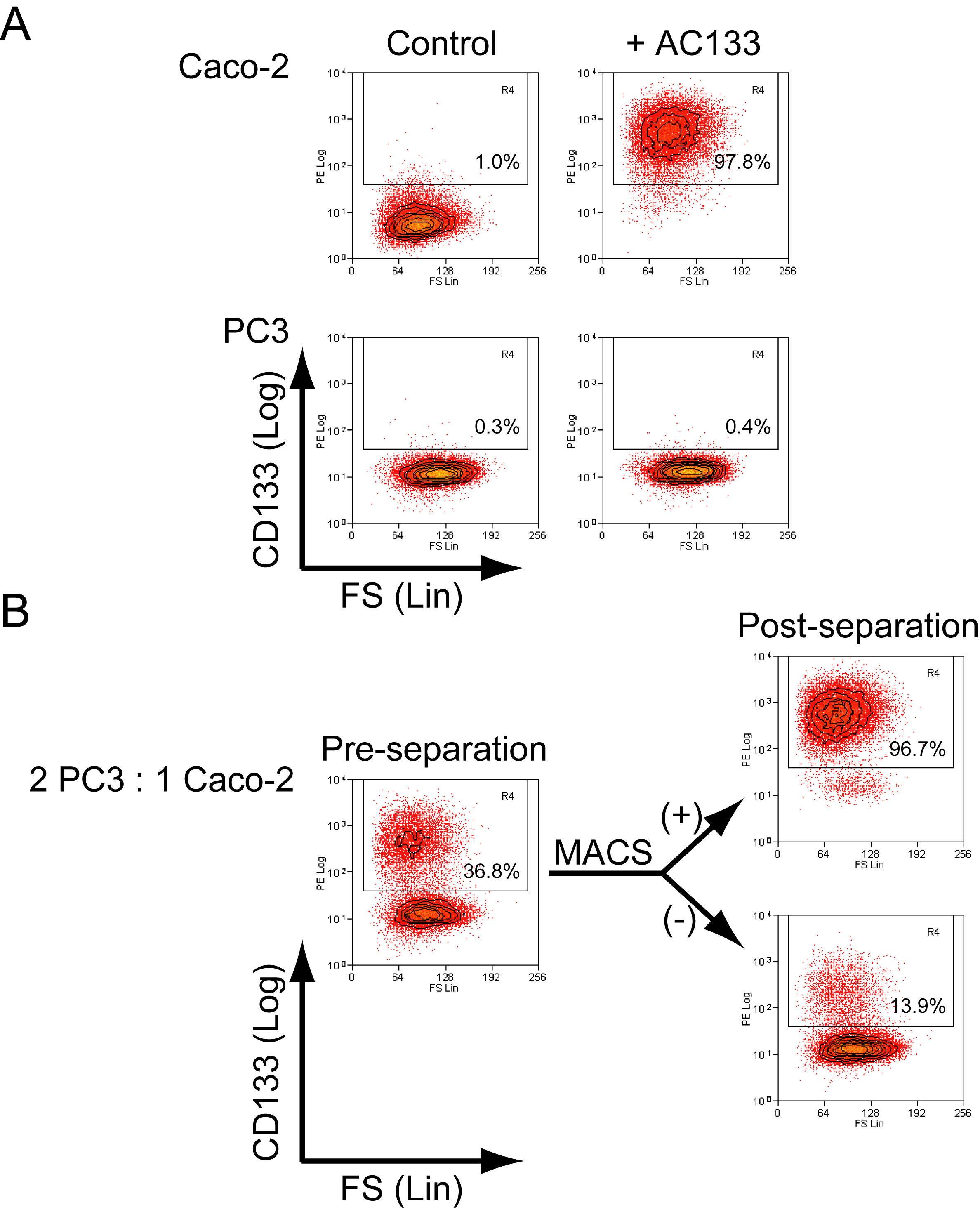

Supplement: Figure S4 — Flow cytometric purity assessment of positive and negative cell fractions following immunomagnetic separation of a mixture of Caco-2 and PC3 cells using CD133 (AC133) antibody (representative images of n = 3). A. Flow cytometric analysis of unsorted Caco-2 and PC3 cells. Almost all Caco-2 cells express CD133 (>97%), in contrast to PC3 cells which were CD133− by phenotype. B. The two cell lines were mixed at a ratio of 2 PC3 : 1 Caco-2 and sorted by MACS to obtain positive and negative fractions. Flow cytometric purities of CD133+ and CD133− fractions post-selection were 95.4±3.21% and 81.7±5.40%, respectively (n = 3). n = 20,000 live cells for all analyses. FS = forward scatter. MACS = magnetic cell separation (TIF) [file pone.0046979.s004.tif]

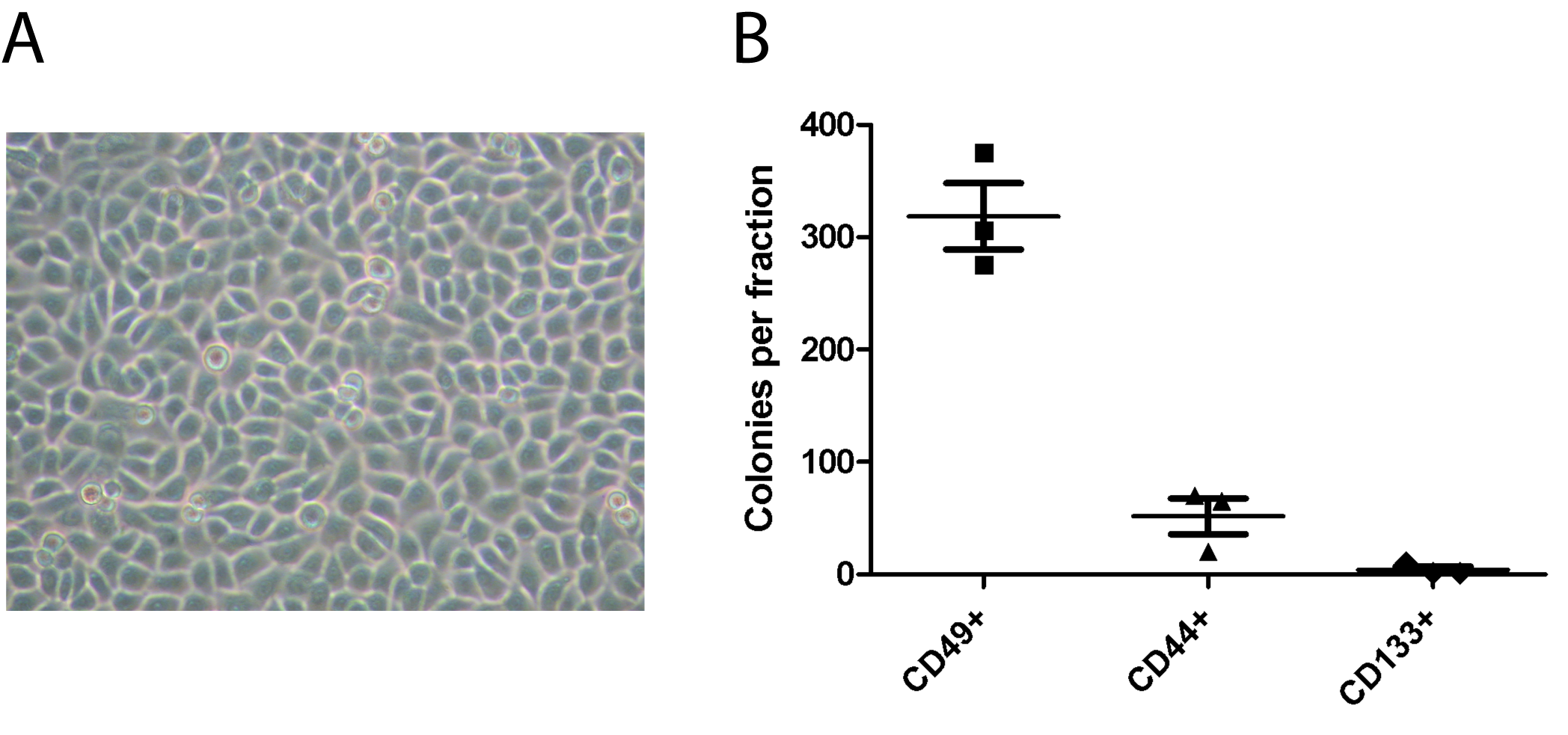

Supplement: Figure S5 — A. Human prostate epithelial cells can be cultured without collagen coating (a representative image of an epithelial colony is shown using the same protocol described in the methods, but without collagen pre-coating). B. CD49f is an integrin with potential to bind collagen [54]. To determine whether collagen-coating was responsible for the enhanced colony forming capacity of CD49f+ cells, the colony-forming assay was repeated as described in Figure 3, without collagen pre-coating. In the absence of collagen coating, our results again showed the highest colony-forming cell recovery in CD49f+ cells. (TIF) [file pone.0046979.s005.tif]
