# Supplementary material for: CD49f Is an Efficient Marker of Monolayer- and Spheroid Colony-Forming Cells of the Benign and Malignant Human Prostate
Source: PLoS One. 2012 Oct 12;7(10):e46979. doi: 10.1371/journal.pone.0046979 (PMC3470557; doi:10.1371/journal.pone.0046979)
Supplement: Table S1 — Table indicating the sources of prostate tissue, patient age, and PSA value for each benign tissue. All samples listed were histologically confirmed to have benign histology. HoLEP = Holmium laser enucleation of prostate. (DOC) [file pone.0046979.s006.doc]

| **Patient**  **Number** | **Procedure** | **Age (yr)** | **PSA (µg/L)** |
| --- | --- | --- | --- |
| **R1** | HoLEP | 56 | 4.3 |
| **R2** | HoLEP | 58 | n/a |
| **R3** | HoLEP | 65 | n/a |
| **R4** | HoLEP | 66 | n/a |
| **R6** | HoLEP | 59 | 5.5 |
| **R8** | HoLEP | 67 | n/a |
| **R9** | HoLEP | 75 | 10 |
| **R10** | HoLEP | 59 | 10.9 |
| **R12** | HoLEP | 66 | 6.55 |
| **R13** | HoLEP | 83 | n/a |
| **R14** | HoLEP | 69 | 8 |
| **R15** | HoLEP | 68 | 18 |
| **R16** | HoLEP | 85 | n/a |
| **R17** | HoLEP | 65 | n/a |
| **R18** | HoLEP | 69 | n/a |
| **R19** | HoLEP | 57 | 5.7 |
| **R20** | HoLEP | 59 | 2.3 |
| **R22** | HoLEP | 50 | 11.8 |
| **R23** | HoLEP | 78 | n/a |
| **R24** | HoLEP | 65 | 4.8 |
| **R26** | HoLEP | 72 | 8.0 |
| **R27** | HoLEP | 80 | n/a |
| **Mean** | 66.9 | 90.9 | 8.0 |
| **S.D.** | 9.2 | 37.9 | 4.2 |

Table S1.
